# Exploring the molecular mechanisms of macrophages in islet transplantation using single-cell analysis

**DOI:** 10.3389/fimmu.2024.1407118

**Published:** 2024-08-29

**Authors:** Zuhui Pu, Shujuan Chen, Ying Lu, Zijing Wu, Zhiming Cai, Lisha Mou

**Affiliations:** ^1^ Imaging Department, Institute of Translational Medicine, Health Science Center, The First Affiliated Hospital of Shenzhen University, Shenzhen Second People’s Hospital, Shenzhen, Guangdong, China; ^2^ MetaLife Lab, Shenzhen Institute of Translational Medicine, Shenzhen, Guangdong, China; ^3^ Department of Endocrinology, Institute of Translational Medicine, Health Science Center, The First Affiliated Hospital of Shenzhen University, Shenzhen Second People’s Hospital, Shenzhen, Guangdong, China; ^4^ BGI Medical Group, Shenzhen, Guangdong, China; ^5^ Shenzhen Institute of Synthetic Biology, Shenzhen Institutes of Advanced Technology, Chinese Academy of Sciences, Shenzhen, Guangdong, China

**Keywords:** type 1 diabetes (T1D), islet transplantation, macrophages, immune rejection, single-cell RNA sequencing (scRNA-seq), syngeneic transplantation, allogeneic transplantation, cytokine

## Abstract

**Background:**

Islet transplantation is a promising treatment for type 1 diabetes that aims to restore insulin production and improve glucose control, but long-term graft survival remains a challenge due to immune rejection.

**Methods:**

ScRNA-seq data from syngeneic and allogeneic islet transplantation grafts were obtained from GSE198865. Seurat was used for filtering and clustering, and UMAP was used for dimension reduction. Differentially expressed genes were analyzed between syngeneic and allogeneic islet transplantation grafts. Gene set variation analysis (GSVA) was performed on the HALLMARK gene sets from MSigDB. Monocle 2 was used to reconstruct differentiation trajectories, and cytokine signature enrichment analysis was used to compare cytokine responses between syngeneic and allogeneic grafts.

**Results:**

Three distinct macrophage clusters (Mø-C1, Mø-C2, and Mø-C3) were identified, revealing complex interactions and regulatory mechanisms within macrophage populations. The significant activation of macrophages in allogeneic transplants was marked by the upregulation of allograft rejection-related genes and pathways involved in inflammatory and interferon responses. GSVA revealed eight pathways significantly upregulated in the Mø-C2 cluster. Trajectory analysis revealed that Mø-C3 serves as a common progenitor, branching into Mø-C1 and Mø-C2. Cytokine signature enrichment analysis revealed significant differences in cytokine responses, highlighting the distinct immunological environments created by syngeneic and allogeneic grafts.

**Conclusion:**

This study significantly advances the understanding of macrophage roles within the context of islet transplantation by revealing the interactions between immune pathways and cellular fate processes. The findings highlight potential therapeutic targets for enhancing graft survival and function, emphasizing the importance of understanding the immunological aspects of transplant acceptance and longevity.

## 1 Introduction

Islet transplantation, a promising treatment for type 1 diabetes (T1D), aims to restore insulin production and achieve better glucose control (1). Although allogeneic islet transplantation has been approved and utilized in several countries for many years, the recent FDA approval of Lantidra in the United States marked a significant milestone in T1D treatment (2–4). According to a 20-year report of islet transplantation, significant progress has been made in improving graft survival and function, with advancements in immunosuppressive protocols and transplantation techniques contributing to better outcomes (5). Despite this progress, long-term graft survival and functionality remain challenging, primarily due to immune rejection (6, 7). Traditional approaches involve immunosuppressants, which have significant side effects, including increased infection and tumor risk (8–10).

The application of single-cell RNA sequencing (scRNA-seq) technology in islet transplantation is particularly novel and urgent due to its unparalleled ability to provide detailed insights into cellular heterogeneity and dynamic gene expression profiles at single-cell resolution. This technology allows us to dissect the complex immune microenvironment within transplanted islets, specifically focusing on macrophages, which play a pivotal role in graft acceptance and rejection. Previous methodologies, such as bulk RNA sequencing, lack the ability to identify distinct cellular subtypes and their specific functions within the graft microenvironment. In contrast, scRNA-seq enables the identification and characterization of diverse macrophage subsets and their roles in modulating immune responses.

The complexity of the immune microenvironment extends beyond the immediate challenges of immune rejection and immunosuppressant usage to include the elaborate interplay between transplanted islet cells and host immune cells, such as T cells (46), B cells, macrophages, dendritic cells, NK cells and neutrophils (11–13). Macrophages are critical in islet transplantation due to their dual role in promoting tissue repair and mediating immune responses (14). These versatile cells are involved in various processes, including phagocytosis, antigen presentation, and cytokine production, which influence graft survival and function. The ability of these cells to polarize into either proinflammatory (M1) or anti-inflammatory (M2) phenotypes significantly influences graft outcomes (15). Previous studies have highlighted the conflicting roles of macrophages in islet transplantation, with some reports indicating their contribution to graft rejection and others suggesting their involvement in immune tolerance. However, these studies were limited by their inability to precisely characterize macrophage subsets and their functional states within the transplant microenvironment.

Recent research underscores the therapeutic potential of macrophages due to their plasticity and diverse functions. Macrophage-based cell therapy can be engineered for tissue repair, immune modulation, and targeting specific diseases (14). Alpha-1 antitrypsin has been shown to suppress proinflammatory macrophage activity, improving islet graft survival (15). Polylysine-bilirubin conjugates support islet viability and promote M2 macrophage polarization, aiding transplant acceptance (16). Islet transplantation can modulate macrophage activity to induce immune tolerance and promote angiogenesis, enhancing transplant success (17). Additionally, immunomodulatory injectable silk hydrogels maintain functional islets and promote M2 macrophage polarization, facilitating graft acceptance (18).

Our previous research identified three distinct macrophage subsets (Mø-C1, Mø-C2, and Mø-C3) through scRNA-seq, revealing their complex involvement in immune rejection and tolerance processes (19). Building on this foundational work, the current study presents a reanalysis of an existing scRNA-seq dataset from mouse transplantation models to characterize macrophage phenotypes associated with syngeneic and allogeneic islet grafts. The aim of this study was to further elucidate the mechanisms by which these macrophages contribute to transplantation outcomes. By comparing key pathways, we sought to uncover the specific roles of macrophage subsets in graft outcomes. This detailed profiling not only enhances our understanding of macrophage biology in islet transplantation but also identifies potential therapeutic targets to improve transplant success and longevity.

## 2 Materials and methods

### 2.1 Single-cell data analysis of islet grafts

ScRNA-seq data of syngeneic islet transplantation and allogeneic islet transplantation grafts were obtained from GSE198865 (19). Seurat (version 4.4.0) was used for filtering and subsequent clustering (20). Cells with RNA feature counts less than 200 or greater than 4500 and a mitochondrial content exceeding 15% were excluded as poor-quality cells. Genes not detected in at least 3 cells were removed from subsequent analysis. These thresholds were set to eliminate low-quality cells and potential doublets, ensuring the reliability of downstream analyses. The mitochondrial content threshold is based on the principle that high mitochondrial gene expression may indicate stressed or dying cells, which could bias the results.

Uniform manifold approximation and projection for dimension reduction (UMAP) (21) was performed using the Seurat R package with the first 75 principal components after performing principal component analysis (PCA) on the 2000 most highly expressed genes. Identification of significant clusters was performed using the FindClusters algorithm in the Seurat package with the resolution set to 0.6. Batch effect correction was performed using the “RunHarmony” function (22). Cell subtypes were annotated according to cell markers from the original study (19).

### 2.2 Differentially expressed genes (DEG) analyzed

For the analysis of DEGs, we used the Wilcoxon rank-sum test for comparisons between two groups. This nonparametric test is suitable for comparing two independent groups and is robust for single-cell RNA sequencing data. The analysis was further refined using the limma package in R (version 4.2.2), where genes were identified as differentially expressed based on two criteria: fold change > 0.25 and an adjusted *P* value < 0.05. Venn diagrams and heatmaps were generated to visualize the interactions between the DEGs and key pathway gene sets. Heatmaps were generated to visualize the results.

### 2.3 Gene set variation analysis (GSVA)

Pathway analyses were predominantly performed on the HALLMARK gene sets described in the Molecular Signatures Database (MSigDB) and exported using the MSigDB package (version 7.5.1). We applied GSVA using standard settings, as implemented in the GSVA package (version 1.46.0) (23). Differences in pathway activity per cell according to GSVA among the different macrophage clusters. To correct for multiple comparisons, we employed the Benjamini-Hochberg method to control the false discovery rate (FDR). This correction is crucial for minimizing type I errors when conducting multiple statistical tests simultaneously.

### 2.4 Analyzing the role of key gene sets in macrophages

To explore the roles of related gene sets in macrophages identified in GSVA, we conduct a specialized analysis of the expression patterns of these gene sets to uncover their potential role in transplant immune responses. Venn diagrams are used to display the intersection genes between each gene set and the DEGs in various macrophage subgroups from both syngeneic and allogeneic transplants.

### 2.5 Reconstruction of differentiation trajectories using Monocle 2

Using the R package Monocle 2 (version 2.8.0) (24), differentiation hierarchies within different clusters were reconstructed. Cell fate decisions and differentiation trajectories were reconstructed with the Monocle 2 package, which utilized reverse graph embedding based on a user-defined gene list to generate a pseudotime plot that could account for both branched and linear differentiation processes.

### 2.6 Cytokine signature enrichment analysis

To assess the cytokine signatures of macrophage subsets (Mø-C1, Mø-C2, and Mø-C3) in syngeneic and allogeneic islet transplantation grafts, we utilized the Dictionary of Immune Responses to Cytokines at single-cell resolution. This approach was based on the transcriptional response data to individual cytokine stimulation collected by Cui et al. (25). We compared the cytokine signatures of macrophage subsets after allogeneic islet transplantation to those after syngeneic islet transplantation. Immune response enrichment analysis (IREA) (25) was subsequently conducted to calculate enrichment scores for each cytokine. This analysis identified the 86 cytokines with the enrichment for each macrophage subset.

### 2.7 Statistical analysis

For the analysis of gene expression in the scRNA-seq data, all single-cell sequencing data statistical analyses were performed in the R Seurat package (version 4.4.0). Heatmaps were generated from the row-scaled expression values using the heatmap package in R (version 4.2.1). We established statistical significance at *P* < 0.05.

## 3 Results

### 3.1 The workflow of this study

**Figure 1:**
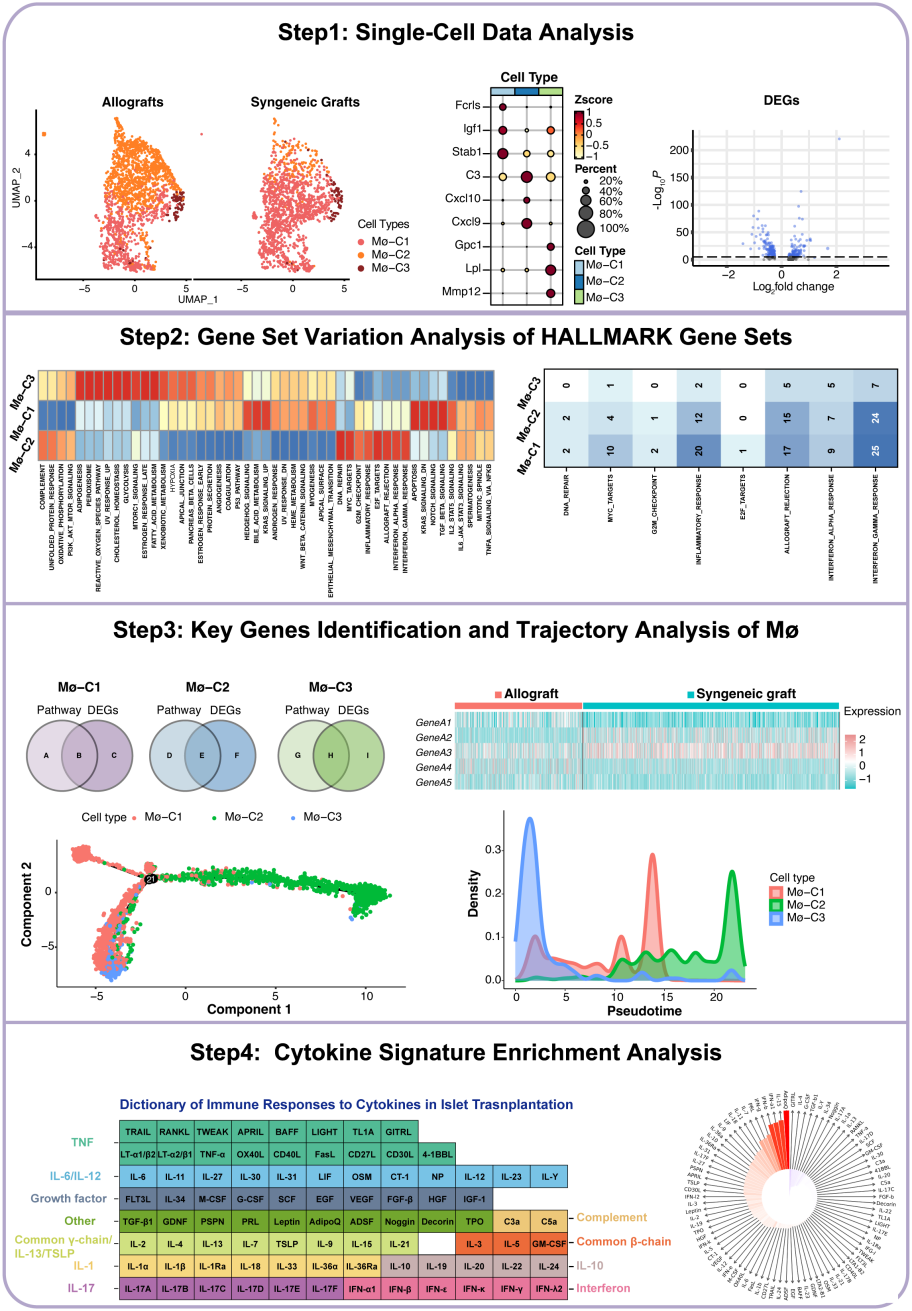
Workflow of this study. Mø, macrophage.

The workflow of this study is illustrated in **Figure 1**. We began by acquiring single-cell datasets from GSE198865 covering both syngeneic and allogeneic islet grafts. Following stringent quality control, normalization, and initial dimensionality reduction, we used uniform manifold approximation and projection (UMAP) to distinguish cellular clusters from syngeneic and allogeneic islet grafts. ScRNA-seq analysis of macrophages revealed three distinct clusters (Mø-C1, Mø-C2, and Mø-C3) with their marker genes. We then conducted differential gene expression analysis across macrophage clusters (Mø-C1, Mø-C2, and Mø-C3) to identify differentially expressed genes (DEGs) between syngeneic and allogeneic grafts. Gene set variation analysis (GSVA) revealed pathway activity differences, with eight pathways upregulated in Mø-C2 macrophages. Intersection analysis identified key genes involved in pathways across Mø-C1, Mø-C2, and Mø-C3, as visualized through Venn diagrams and heatmaps. Trajectory analysis using Monocle 2 and cytokine signature enrichment analysis further elucidated macrophage dynamics and immune responses in islet transplantation.

### 3.2 Analysis of single-cell datasets

**Figure 2:**
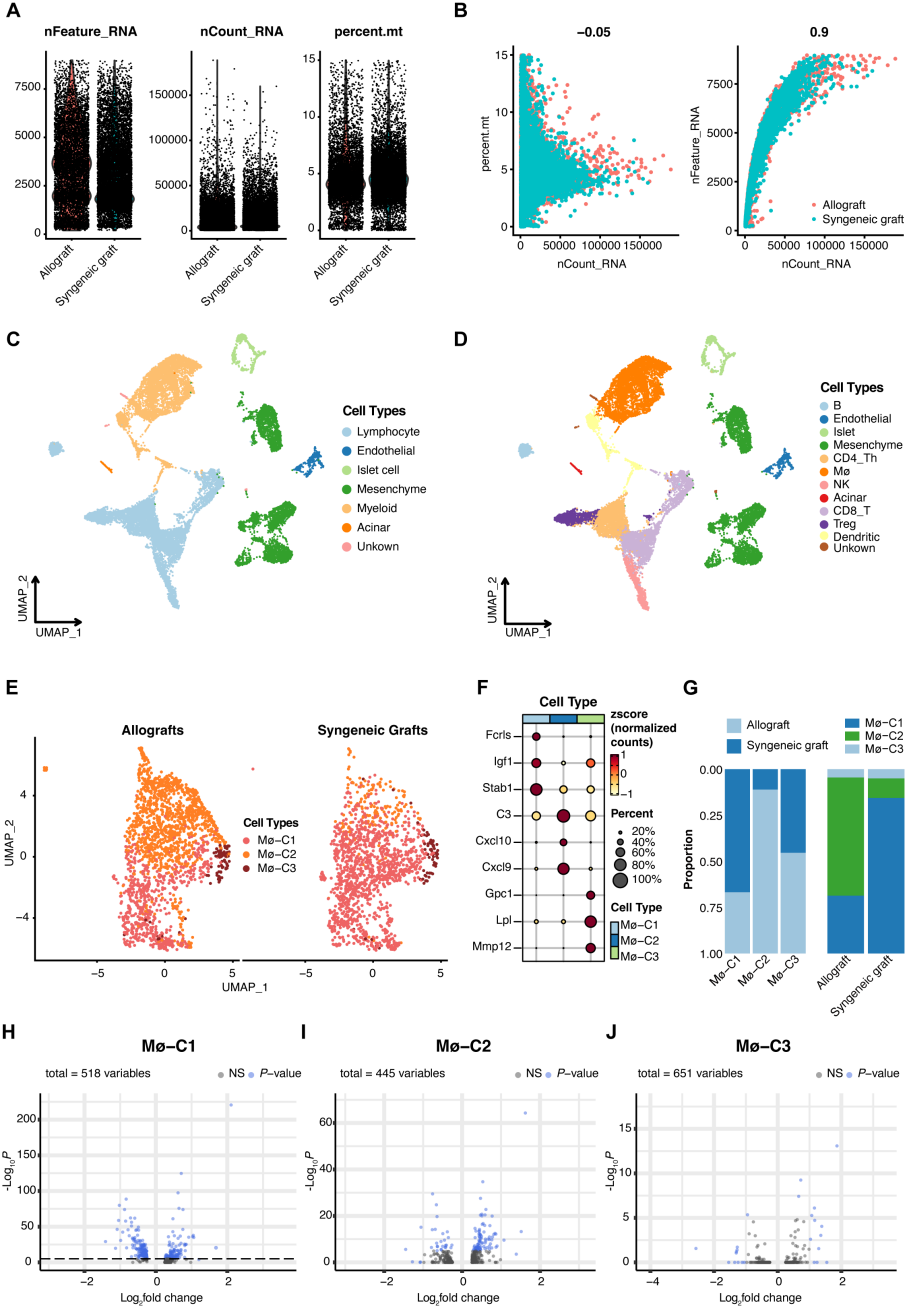
Single-cell RNA sequencing (scRNA-seq) insights into islet transplantation. **(A)** Quality control metrics for scRNA-seq of syngeneic and allogeneic islet grafts. **(B)** The number of detected genes showed no correlation with the percentage of mitochondrial content but was significantly correlated with sequencing depth. **(C)** Uniform manifold approximation and projection (UMAP) visualization highlights six predominant cell types within islet grafts, underscoring the diverse cellular landscape. **(D)** Further UMAP analysis revealed 11 subcell types, providing a detailed view of cellular diversity within the grafts. **(E)** Comparative UMAP plots of three macrophage clusters (Mø-C1, Mø-C2, and Mø-C3) in allogeneic (left panel) versus syngeneic (right panel) islet grafts reveal distinct cellular distributions. **(F)** Maker genes of three macrophage clusters. **(G)** The proportions of Mø-C1, Mø-C2, and Mø-C3. The number of Mø-C2 cells in allografts was significantly greater than that in syngeneic grafts. **(H–J)** The variance in gene expression between syngeneic and allogeneic grafts across macrophage clusters (Mø-C1, Mø-C2, and Mø-C3) is depicted, emphasizing the differential expression landscape. Mø, macrophage.

In the foundational stage of our study, we accessed single-cell datasets encompassing both syngeneic and allogeneic islet grafts sourced from GSE198865 (19). After implementing rigorous quality control measures (**Figures 2A, B**), normalization processes, and initial dimensionality reduction steps, we utilized UMAP for dimension reduction. Cellular gene markers from the original dataset were used to categorize six primary cell types (**Figure 2C**): :lymphocytes(markers: *Cd3e, Cd4* and *Cd8*),endothelial cells (markers: *Pecam1, Egfl7* and *Plvap*), islet cells (markers: *Ins1, Chga* and *Scg2*), mesenchymal cells (markers: *Col3a1, Col1a1* and *Col1a2*), myeloid cells (markers: *Cd68, Gzma* and *Cd7*), and acinar cells (markers: *Amy2a, Ptf1a* and *Mist1*). These major cell types were further partitioned into 11 subcell types (**Figure 2D**): B cells (markers: *Cd19, Cd79a* and *Ms4a1*), endothelial cells, islet cells, mesenchymal cells, CD4+ Th cells (markers: *Cd4, Tnfsf8* and *Lat*), CD8+T cells (markers: *Cd8a, Cd8b1* and *Ms4a4b*), regulatory T cells (Tregs, markers: *Il2ra, Ctla4* and *Cd2*), macrophages (markers: *Cd68, Csf1r* and *Pla2g7*), natural killer cells (NK, markers: *Gzma, Cd7* and *Klrb1c*), acinar cells and dendritic cells (DCs, markers: *Clec9a, Xcr1* and *Cd24a*).

Meticulous scRNA-seq analysis of macrophage populations revealed three transcriptionally unique clusters, namely, Mø-C1, Mø-C2, and Mø-C3, providing deep insights into the heterogeneity and functional specialization of macrophage communities in the context of islet transplantation (**Figure 2E**). The marker genes of Mø-C1, Mø-C2, and Mø-C3 are shown in **Figure 2F**. The proportions of Mø-C1, Mø-C2, and Mø-C3 are shown in **Figure 2G**. The proportion of Mø-C1 cells was significantly greater in syngeneic grafts, whereas the proportion of Mø-C2 cells was considerably greater in allogeneic grafts. The proportion of Mø-C3 cells was similar in both the syngeneic and allogeneic grafts. This granular view of cellular landscapes sets the stage for a nuanced understanding of the immunological intricacies governing graft survival and acceptance.

### 3.3 Comparative analysis of DEGs in macrophages between syngeneic and allogeneic islet transplants

To investigate the molecular differences between macrophages from syngeneic versus allogeneic islet grafts, we conducted a thorough differential gene expression analysis. This approach enabled us to identify and characterize the DEGs across three distinct clusters of macrophages: Mø-C1 (**Figure 2H**, **Supplementary Table 1**), Mø-C2 (**Figure 2I**, **Supplementary Table 2**), and Mø-C3 (**Figure 2J**, **Supplementary Table 3**). By employing bioinformatics tools, we generated volcano plots to visually represent the DEGs between the syngeneic and allogeneic islet grafts within each macrophage subset. The analysis revealed significant differences in the expression of genes involved in critical pathways associated with graft acceptance, immune response modulation, and islet cell survival.

### 3.4 Key pathways upregulated in Mø-C2 macrophages during islet-allograft transplantation

**Figure 3:**
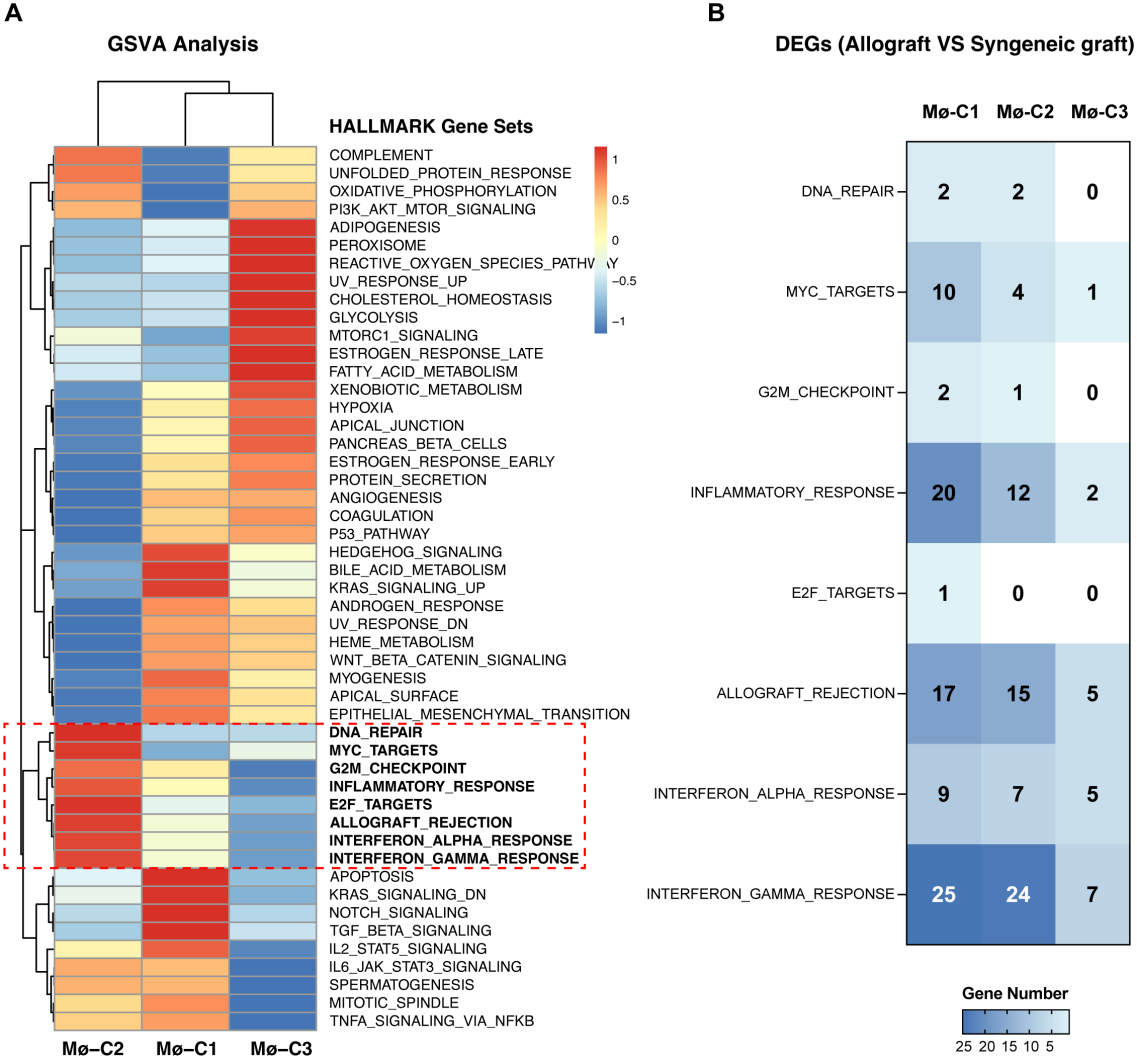
Gene set variation analysis (GSVA). **(A)** Pathway analyses primarily utilized HALLMARK gene sets from the Molecular Signatures Database (MSigDB), which were exported via the MSigDB package. GSVA scores per cell revealed pathway activity differences across macrophage clusters (Mø-C1, Mø-C2, and Mø-C3). The red box highlights eight pathways significantly upregulated in Mø-C2 cells, underscoring their critical role in islet-allograft transplantation. These pathways included DNA repair, MYC target, G2M checkpoint, inflammatory response, E2F target, allograft rejection, interferon alpha response, and interferon gamma response pathways. **(B)** The number of differentially expressed genes (DEGs) within pathways significantly upregulated in Mø-C2 macrophages. Mø, macrophage.

Pathway analyses primarily utilized HALLMARK gene sets from the Molecular Signatures Database (MSigDB), which are exported via the MSigDB package. GSVA scores per cell revealed pathway activity differences across macrophage clusters (Mø-C1, Mø-C2, and Mø-C3). Notably, eight pathways were significantly upregulated in Mø-C2 cells, underscoring their critical role in islet-allograft transplantation. These pathways included DNA repair, MYC targets, G2M checkpoint, inflammatory response, E2F targets, allograft rejection, interferon alpha response, and interferon gamma response (**Figure 3A**). The number of DEGs within these pathways is detailed in **Figure 3B**.

### 3.5 Signaling pathway dynamics in Mø-C1

**Figure 4:**
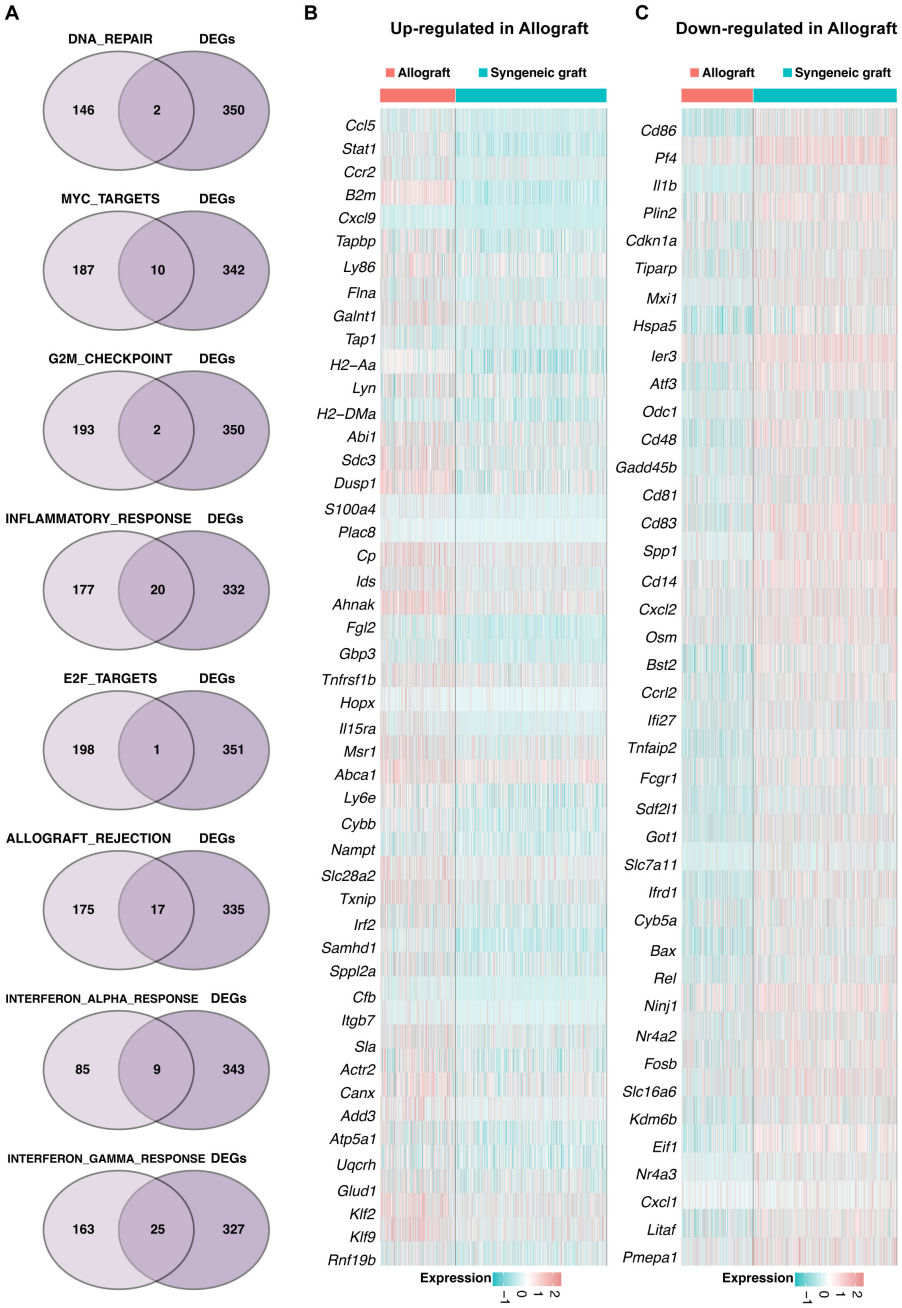
Signaling pathway dynamics in Mø-C1. **(A)** Intersection analysis highlights key genes involved in eight distinct pathways within the Mø-C1 macrophage cluster, as shown in the Venn diagram. These pathways included DNA repair, MYC target, G2M checkpoint, inflammatory response, E2F target, allograft rejection, interferon alpha response, and interferon gamma response pathways. **(B)** Heatmap of up-regulated DEGs in allograft involved in these pathways. **(C)** Heatmap of down-regulated DEGs in allograft involved in these pathways. Mø, macrophage.

Intersection analysis identified key genes involved in eight distinct pathways within the Mø-C1 macrophage cluster, as illustrated by the Venn diagram (**Figure 4A**). These pathways include DNA repair (including 2 genes), MYC targets (including 10 genes), G2M checkpoint (including 2 genes), inflammatory response (including 20 genes), E2F targets (including 1 gene), allograft rejection (including 17), interferon alpha response (including 9 genes), and interferon gamma response (including 25 genes). The heatmaps shows the up-regulated DEGs (**Figure 4B**) and down-regulated DEGs (**Figure 4C**) in allograft compared with syngeneic graft within these pathways. Detailed information on all DEGs in pathways related to Mø-C1 is provided in **Supplementary Tables 4**, **5**. This analysis highlights the involvement of diverse genes in crucial pathways, shedding light on the multifaceted roles of Mø-C1 macrophages in islet grafts.

### 3.6 Pathway analysis in Mø-C2

**Figure 5:**
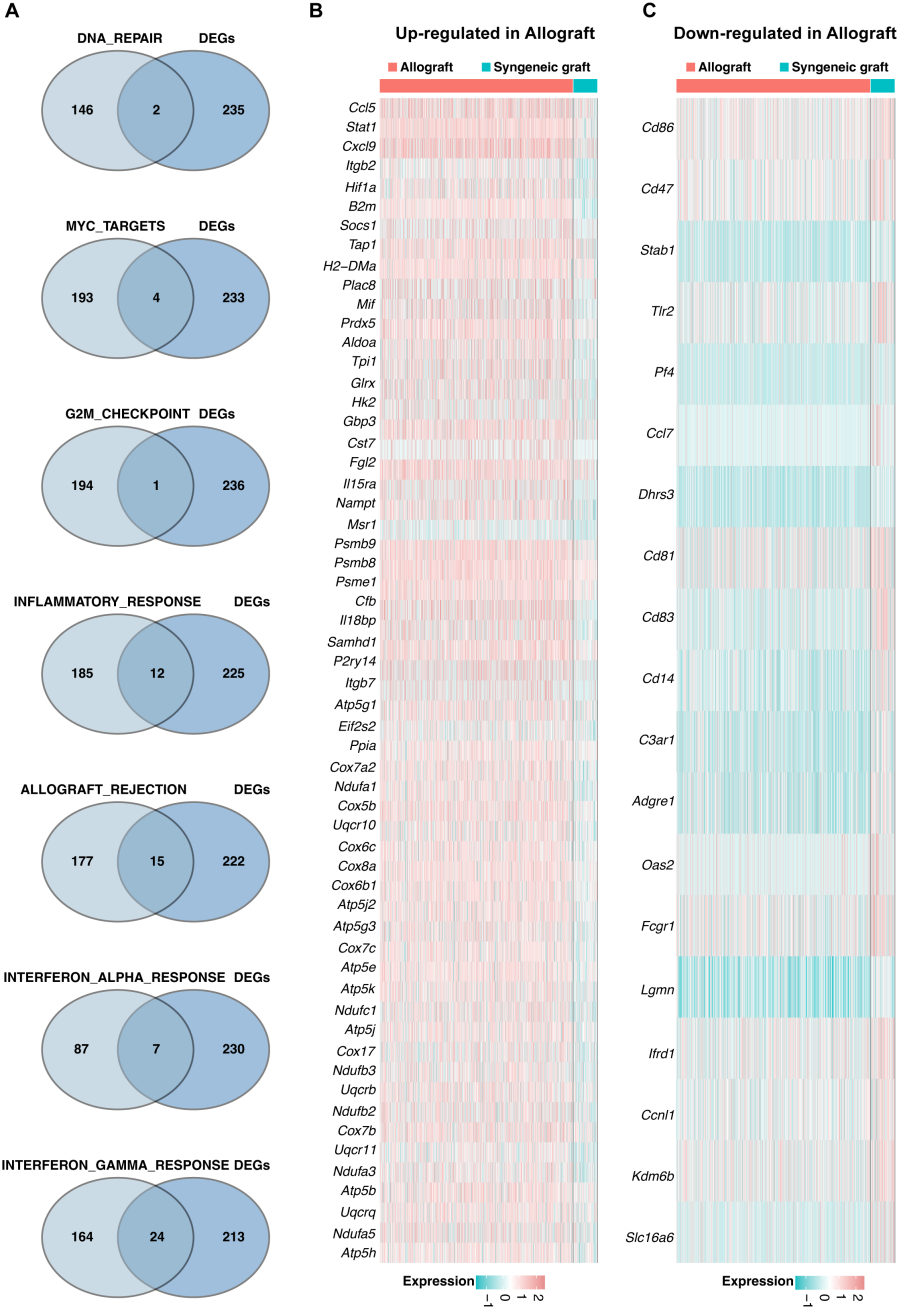
Pathway analysis in Mø-C2 macrophages. **(A)** Venn diagram identifying significant genes across seven pathways within the Mø-C2 cluster. These pathways include DNA repair, MYC targets, G2M checkpoint, inflammatory response, allograft rejection, interferon alpha response, and interferon gamma response pathways. **(B)** The heatmap visualizes the up-regulated DEGs in allograft involved in these pathways. **(B, C)** The heatmap visualizes the down-regulated DEGs in allograft involved in these pathways. Mø, macrophage.

A Venn diagram (**Figure 5A**) was generated to identify genes significantly enriched in seven pathways within the Mø-C2 cluster. These pathways included DNA repair (including 2 genes), MYC targets (including 4 genes), G2M checkpoint (including 1 gene), inflammatory response (including 12 genes), allograft rejection (including 15 genes), interferon alpha response (including 7 genes), and interferon gamma response (including 24 genes). The heatmaps was generated to visualize the up-regulated DEGs (**Figure 5B**) and down-regulated DEGs (**Figure 5C**) in these pathways. Detailed information on all DEGs in pathways related to Mø-C2 is provided in **Supplementary Tables 4**, **5**. This analysis underscores the significant activation of inflammatory and immune response pathways in Mø-C2 macrophages, particularly in the context of allograft rejection.

### 3.7 Pathway insights for Mø-C3 macrophages

**Figure 6:**
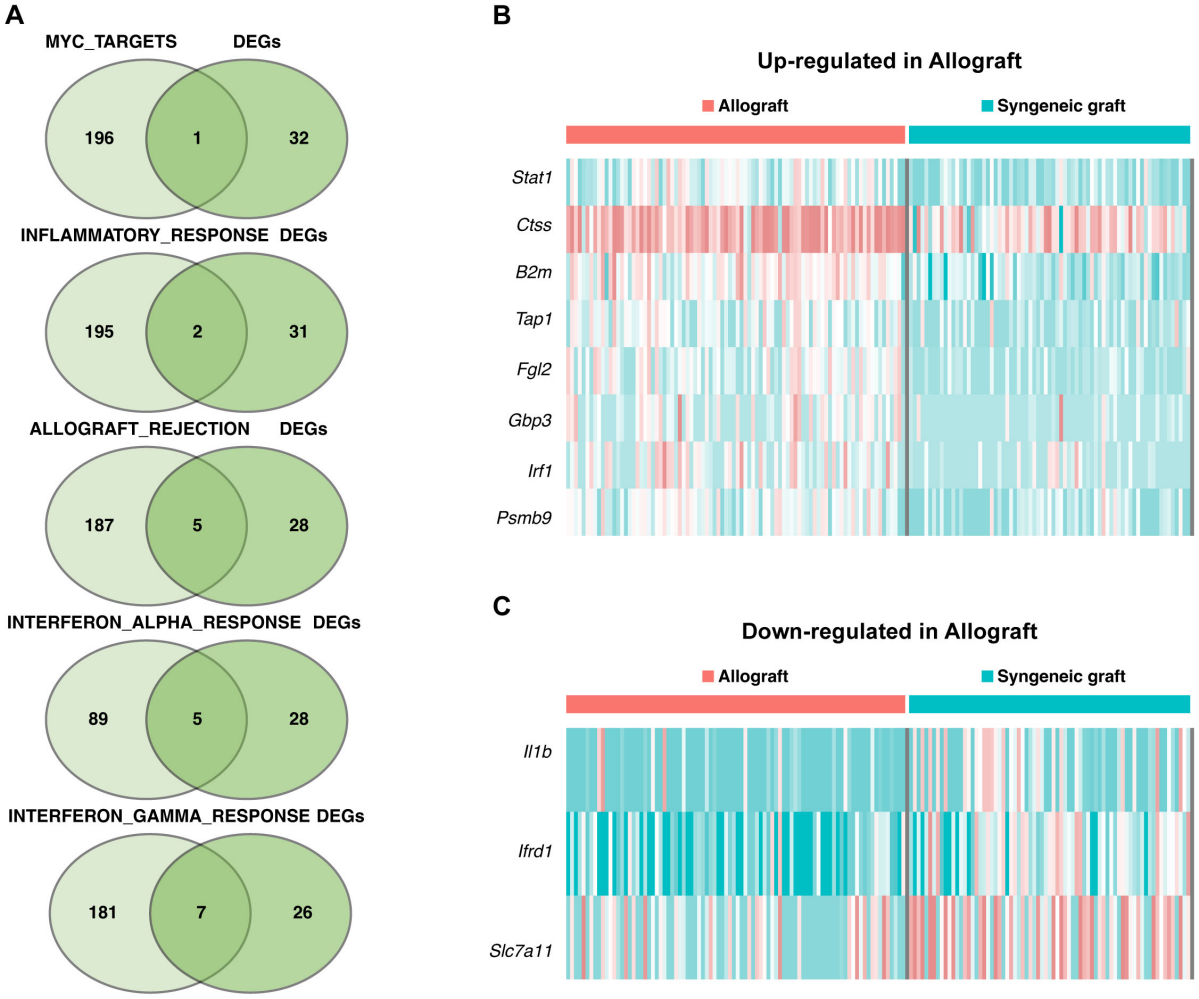
Pathway insights for Mø-C3 macrophages. **(A)** Key genes associated with five pathways in the Mø-C3 cluster were identified through intersection analysis. These pathways include MYC targets, inflammatory response, allograft rejection, interferon alpha response, and interferon gamma response. **(B)** Heatmap showing the up-regulated DEGs in allograft within these pathways. **(C)** Heatmap showing the down-regulated DEGs in allograft within these pathways. Mø, macrophage.

Key genes associated with five pathways in the Mø-C3 cluster were identified through intersection analysis, as shown in the Venn diagram (**Figure 6A**). These pathways included MYC targets (including 1 gene), inflammatory response genes (including 2 genes), allograft rejection genes (including 5 genes), interferon alpha response genes (including 5 genes), and interferon gamma response genes (including 7 genes). The heatmaps shows the up-regulated DEGs (**Figure 6B**) and down-regulated DEGs (**Figure 6C**) within these pathways. Detailed information on all DEGs in pathways related to Mø-C3 cells is provided in **Supplementary Tables 4**, **5**. This analysis revealed the significant roles of the interferon response and allograft rejection pathways in Mø-C3 macrophages, contributing to the understanding of their function in the immune response to transplantation.

### 3.8 Macrophage transcriptional state bifurcation and cell fate of three clusters (Mø-C1, Mø-C2, and Mø-C3)

**Figure 7:**
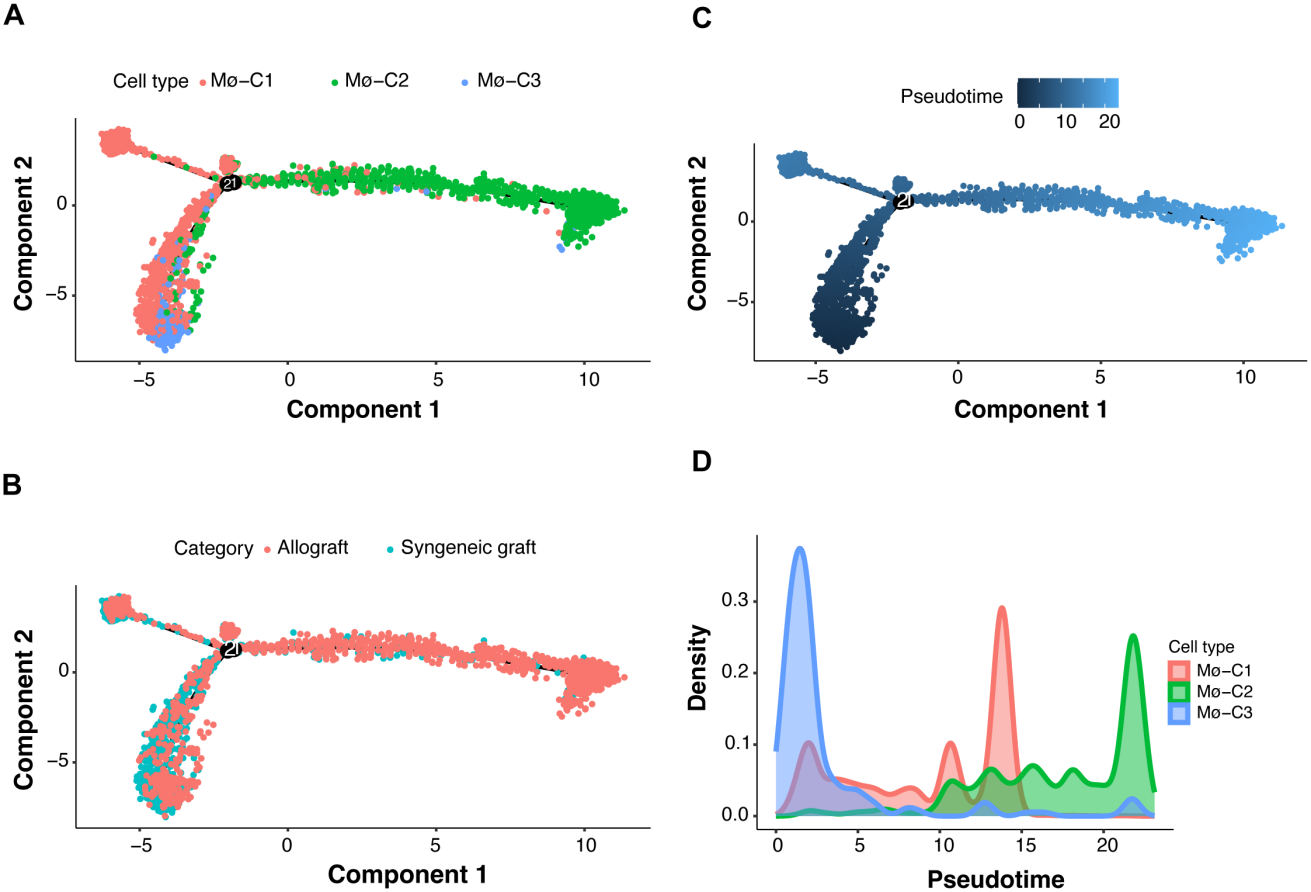
Macrophage transcriptional state bifurcation and cell fate of three clusters (Mø-C1, Mø-C2, and Mø-C3). **(A)** Multifold trajectories of macrophages from islet grafts analyzed using the Monocle 2 algorithm. Solid and dotted lines indicate distinct cellular trajectories or fates as determined by their expression profiles. **(B)** Comparative trajectory analysis of macrophages from syngeneic and allogeneic grafts using the Monocle 2 algorithm. **(C, D)** Density plots illustrating pseudotime projections of transcriptional changes for the three macrophage clusters (Mø-C1, Mø-C2, and Mø-C3). Mø, macrophage.

Trajectory manifold analysis of macrophages from islet grafts was conducted using the Monocle 2 algorithm, which identified distinct cellular trajectories or fates based on expression profiles (**Figure 7A**). The analysis revealed that macrophages primarily originate from the Mø-C3 cluster, which branches into the Mø-C1 and Mø-C2 clusters. Comparative trajectory analysis of macrophages from syngeneic and allogeneic grafts (**Figure 7B**) further elucidated these dynamics, showing that Mø-C3s serve as common progenitors for both transplant types.

The density plots (**Figures 7C, D**) illustrate the pseudotime projections of transcriptional changes for the three macrophage clusters (Mø-C1, Mø-C2, and Mø-C3). The proportion of Mø-C3 macrophages was relatively similar in both syngeneic and allogeneic grafts (**Figure 2G**), indicating that a stable progenitor state was unaffected by the type of graft. However, Mø-C1 macrophages were found in greater proportions in syngeneic grafts (**Figure 2G**), suggesting that they play a role in promoting graft tolerance. Conversely, Mø-C2 macrophages were more prevalent in allogeneic grafts (**Figure 2G**), which is indicative of their involvement in inflammatory responses and graft rejection.

### 3.9 Cytokine signature enrichment in macrophages

**Figure 8:**
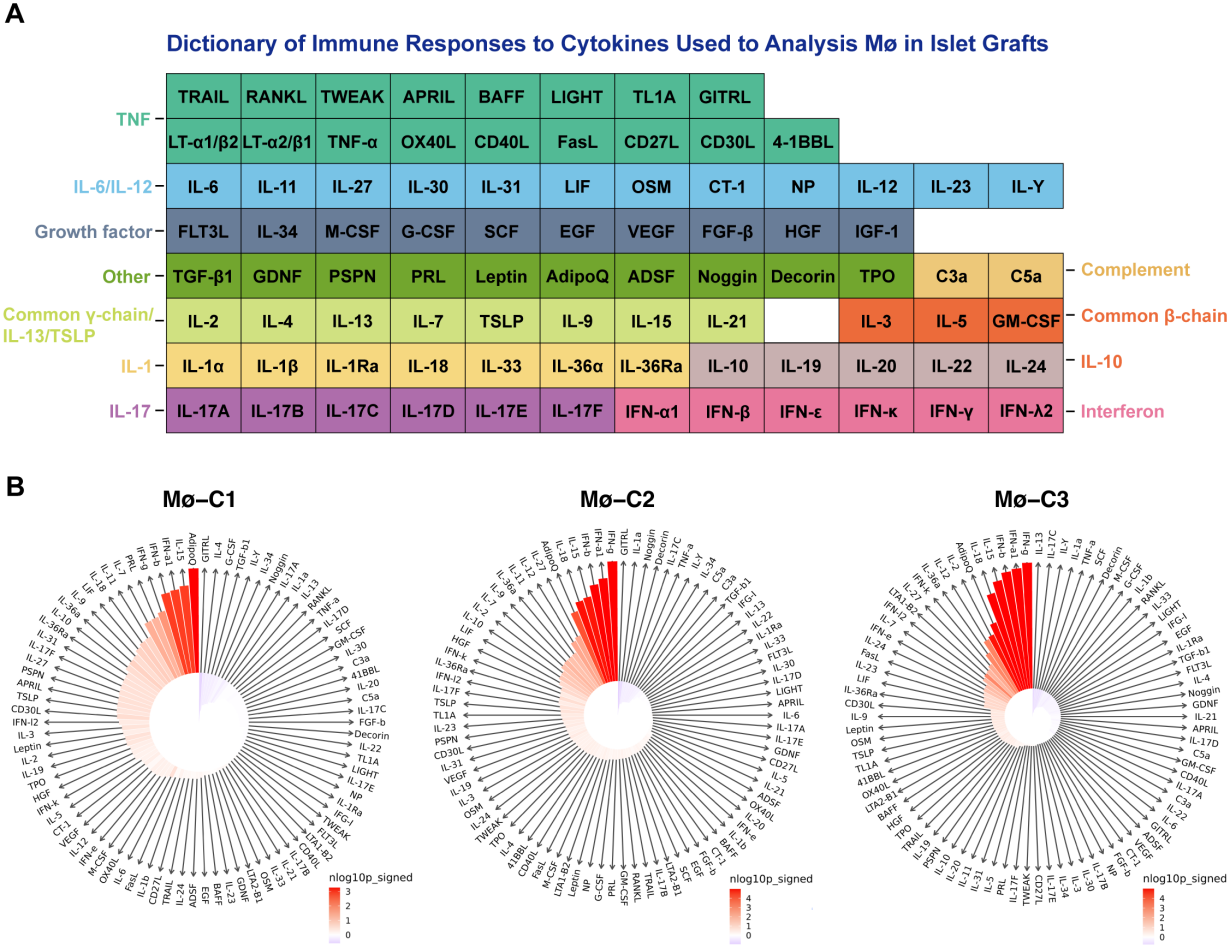
Cytokine signature enrichment in macrophages. **(A)** A comprehensive dictionary of immune responses to cytokines was utilized to evaluate macrophages in islet grafts. We analyzed the responses to 86 cytokines by comparing syngeneic and allogeneic grafts. **(B)** The IREA cytokine enrichment plot displays the enrichment score (ES) for each of the 86 cytokine responses across three macrophage clusters (Mø-C1, Mø-C2, and Mø-C3) in allogeneic versus syngeneic grafts. The length of the bars indicates the ES, while the shading reflects the FDR-adjusted P value from a two-sided Wilcoxon rank-sum test, with darker shades denoting greater statistical significance (red for allografts, blue for syngeneic grafts). Mø, macrophage.

To evaluate macrophages in islet grafts, we employed a comprehensive dictionary of immune responses to cytokines. Responses to 86 cytokines were analyzed by comparing syngeneic and allogeneic grafts (**Figure 8A**). The immune response enrichment analysis (IREA) cytokine enrichment plot (**Figure 8B**) displays the enrichment score (ES) for each cytokine response across the three macrophage clusters (Mø-C1, Mø-C2, and Mø-C3) in syngeneic versus allogeneic grafts. The bar length represents the ES, while shading indicates the FDR-adjusted *P* value from a two-sided Wilcoxon rank-sum test, with darker shades reflecting greater statistical significance (red for allografts, blue for syngeneic grafts).

This evaluation is based on data collected by Cui et al. (25), where transcriptional responses to individual cytokine stimulation were measured. For Mø-C1s, the top 10 cytokines with the strongest enrichment in allografts compared to syngeneic grafts were adiponectin, IL15, IFNα1, IFNβ, IFNγ, prolactin, IL7, IL11, IL18, and LIF (detailed results are provided in **Supplementary Table 6**). For Mø-C2s, the top 10 cytokines with the strongest enrichment in allografts were IFNγ, IFNα1, IFNβ, IL15, IL18, adiponectin, IL27, IL12, IL11, and IL36α (detailed results are provided in **Supplementary Table 7**). For Mø-C3s, the top 10 cytokines with the strongest enrichment in allografts were IFNγ, IFNα1, IFNβ, IL15, IL18, adiponectin, IL2, IL12, IL36α, and IFNκ (detailed results are provided in **Supplementary Table 8**).

These analyses collectively offer a comprehensive view of the molecular underpinnings that define the macrophage-mediated response in syngeneic and allogeneic islet transplantation. By shedding light on the specific pathways and genes differentially expressed in various macrophage populations, this research underscores the complexity of the immune response to transplantation and points toward potential therapeutic targets for enhancing graft survival and function.

## 4 Discussion

Single-cell RNA sequencing (scRNA-seq) technology has already found extensive applications in immunology (26) and transplantation (27) research due to its ability to provide high-resolution insights into cellular heterogeneity and the distinct functional states of individual cells. In this study, we leveraged single-cell RNA sequencing to thoroughly examine macrophage dynamics and molecular mechanisms in islet transplantation by comparing syngeneic and allogeneic grafts. By analyzing data from GSE198865 (19), we identified three distinct macrophage clusters (Mø-C1, Mø-C2, and Mø-C3) and explored their differential gene expression and pathway activities. Our detailed single-cell analysis revealed complex interactions and regulatory mechanisms within macrophage populations that were not previously captured by bulk RNA sequencing studies. This detailed view of cellular heterogeneity and functional specialization provides deeper insights into the molecular underpinnings of immune responses in transplantation, thereby the existing research.

Recent studies have demonstrated that macrophage heterogeneity and the distinct functional roles of various macrophage subpopulations are critical in shaping immune responses in different tissue contexts (28). For instance, research has shown that tissue-resident macrophages exhibit unique gene expression profiles and functional specializations depending on their tissue of origin and local microenvironment (29). Furthermore, the diversity of macrophage activation states, ranging from proinflammatory to anti-inflammatory and tissue repair phenotypes, underscores the complexity of their roles in immune regulation (30).

By comparing our findings with those in the literature, we observe both the confirmation and expansion of previously reported results. The observed upregulation of allograft rejection, inflammatory response (31, 32), and interferon (33) signaling pathways in allogeneic transplants corroborates previous studies emphasizing the central role of these pathways in mediating inflammatory responses and immune rejection. However, our study extends these findings by providing a more nuanced understanding of the differences in macrophage polarization states between syngeneic and allogeneic transplants, highlighting a skew toward a more inflammatory phenotype in allogeneic settings. Our analysis revealed significant activation of macrophages in allogeneic transplants, marked by the upregulation of allograft rejection-related genes and pathways involved in inflammatory and interferon responses, supporting the hypothesis that immune rejection in allogeneic transplants is driven by the host’s immune response to foreign antigens.

Gene set variation analysis (GSVA) is a nonparametric, unsupervised method that assesses pathway activity changes over a sample population in an expression dataset. GSVA transforms gene expression data from a gene-centric to a pathway-centric view, enabling the evaluation of pathway-level changes across samples (23). It has been widely utilized in immunological studies to elucidate the involvement of various signaling pathways in immune responses, disease mechanisms, and therapeutic interventions, providing insights into the functional context of gene expression alterations in immune cells (34). We used GSVA methods and identified eight pathways that were significantly upregulated in the Mø-C2 cluster, namely, DNA repair, MYC target, G2M checkpoint, inflammatory response, E2F target, allograft rejection, interferon alpha response, and interferon gamma response pathways. These pathways are crucial for understanding the immune response dynamics in islet transplantation and highlight potential therapeutic targets to modulate macrophage activity and improve graft outcomes.

Detailed intersection analyses and heatmaps of differentially expressed genes (DEGs) across the macrophage clusters provided insights into the polarization states of macrophages. These findings suggest a shift toward a proinflammatory phenotype in allogeneic transplants, which may contribute to graft rejection. This insight is crucial for developing targeted therapies that could reprogram macrophages to a more tolerogenic state.

Monocle 2, originally described by Qiu et al., utilizes a technique called reverse graph embedding to reconstruct the trajectories of single cells as they progress through different states (24). This method is particularly powerful for revealing the dynamic changes in cell fate decisions over time. Monocle 2 constructs a trajectory of single-cell transcriptomes by ordering cells along a pseudotime axis, which helps in understanding the progression and differentiation of cells in various biological processes.

Recent studies have demonstrated the application of Monocle 2 in various research contexts. For instance, Wang et al. (35) used Monocle 2 to create a single-cell transcriptome atlas of human euploid and aneuploid blastocysts, providing insights into early human development and chromosomal abnormalities. Huang et al. (36) applied Monocle 2 to explore the molecular landscape of sepsis severity in infants, revealing that enhanced coagulation, innate immunity, and T-cell repression are key factors. Additionally, Su et al. (37) conducted a direct comparison of mass cytometry and single-cell RNA sequencing of human peripheral blood mononuclear cells using Monocle 2 to elucidate cellular heterogeneity and immune responses. Walzer et al. (38) employed Monocle 2 to study the transcriptional control of the Cryptosporidium life cycle, shedding light on the parasite’s developmental stages and potential therapeutic targets. Furthermore, Wu et al. (39) integrated single-cell sequencing and bulk RNA-seq to identify and develop a prognostic signature related to colorectal cancer stem cells, utilizing Monocle 2 to trace the differentiation pathways of cancer stem cells. These applications highlight Monocle 2’s versatility and effectiveness in tracing cell fate decisions and understanding complex biological processes, making it a valuable tool in both basic and translational research.

Trajectory analysis using Monocle 2 revealed distinct cellular trajectories and fate decisions within macrophage populations, further elucidating the complexity of macrophage responses in islet grafts. The analysis showed that Mø-C3 serves as a common progenitor, branching into Mø-C1 and Mø-C2. Interestingly, the proportion of Mø-C3 cells was similar in both the syngeneic and allogeneic grafts, indicating that the baseline macrophage state was unaffected by the transplant type. In contrast, Mø-C1 cells were predominantly present in syngeneic grafts, while Mø-C2 cells were more abundant in allogeneic grafts. This suggests that Mø-C1 macrophages are more strongly associated with a tolerogenic environment, whereas Mø-C2 macrophages are linked to a more inflammatory response characteristic of graft rejection.

Our cytokine signature enrichment analysis revealed notable differences in cytokine responses between syngeneic and allogeneic grafts. For Mø-C1 macrophages, the top 10 cytokines with the strongest enrichment in allografts compared to syngeneic grafts included adiponectin, IL15, IFNα1, IFNβ, IFNγ, prolactin, IL7, IL11, IL18, and LIF. These cytokines are known to play diverse roles in immune modulation and inflammation. For example, IFNγ (40, 41) and IFNβ (42) are critical for enhancing antigen presentation and promoting a Th1 immune response, which is often associated with graft rejection. Similarly, IL15 and IL18 (43) are potent activators of NK cells and T cells, further contributing to the inflammatory milieu.

In Mø-C2 macrophages, the top 10 enriched cytokines in allografts were IFNγ, IFNα1, IFNβ, IL15, IL18, adiponectin, IL27, IL12, IL11, and IL36α. The presence of IL27 (44) and IL12 (45) suggests their strong involvement in promoting Th1 and Th17 responses, which are crucial for initiating and sustaining immune responses against transplanted tissues. IL36α, a member of the IL-1 cytokine family, is known for its role in amplifying inflammatory responses and has been implicated in autoimmune diseases, suggesting its potential involvement in graft rejection mechanisms.

For Mø-C3 macrophages, the top 10 enriched cytokines in allografts were IFNγ, IFNα1, IFNβ, IL15, IL18, adiponectin, IL2, IL12, IL36α, and IFNκ. IL2 is essential for T-cell proliferation and survival, indicating a supportive environment for effector T-cell responses in allogeneic grafts (45). The enrichment of IFNκ (42), an interferon involved in antiviral responses, further highlights the complexity and multifaceted nature of the immune response in allogeneic grafts.

The enrichment of these cytokines in allografts underscores their critical roles in mediating immune responses and promoting inflammatory environments that are conducive to graft rejection. In contrast, syngeneic grafts, which are genetically identical to the host, do not provoke such robust inflammatory cytokine responses, allowing for better graft acceptance. Our findings align with previous studies showing that proinflammatory cytokines, such as IFNγ, are upregulated in allogeneic transplants, contributing to graft rejection. Conversely, the role of anti-inflammatory cytokines such as IL-10 in supporting graft acceptance is well documented, highlighting their importance in creating a tolerogenic environment in syngeneic transplants.

The distinct cytokine profiles observed in our study highlight the importance of cytokine signaling pathways in shaping the immune landscape during transplantation. Targeting specific cytokines or their signaling pathways could offer new therapeutic strategies to balance immune activation and tolerance, thereby improving graft survival and function. Future research should focus on developing targeted therapies that modulate these cytokine responses to improve transplant outcomes.

While our study provides substantial insights, it is essential to acknowledge several limitations. Primarily, the reliance on animal models necessitates careful consideration when extrapolating findings to human clinical scenarios. Validation in human transplant samples is crucial to ensure clinical relevance. Additionally, single-cell RNA sequencing captures a snapshot of gene expression, which may not fully represent dynamic cellular processes.

Despite rigorous statistical methods, including the Wilcoxon rank-sum test and the limma package, there remains significant variability in gene expression profiles across different macrophage clusters and transplantation models. Differences in cell capture efficiency, sequencing depth, and batch effects can introduce biases, despite stringent quality control measures and batch effect correction.

Future research should focus on corroborating these insights in human transplant samples and exploring the therapeutic potential of targeting identified pathways to modulate macrophage function and improve transplant efficacy. Exploring immune regulatory strategies that specifically target the proinflammatory macrophage response while avoiding broad immunosuppression represents a promising research direction. Finally, experimental validation of computational predictions, such as key pathway activation and cytokine expression, is essential to corroborate our results and translate them into clinical applications. Addressing these limitations in future studies will be critical for advancing our understanding of macrophage dynamics in islet transplantation and improving clinical outcomes.

In conclusion, our study significantly advances the knowledge of macrophage roles within the context of islet transplantation. By meticulously dissecting the interactions between immune pathways and cellular fate processes, we provide a detailed understanding of the immune response and identify potential targets for therapeutic intervention. These findings lay a foundation for innovative research pathways and therapeutic strategies aimed at improving transplantation therapies and achieving long-term success in treating type 1 diabetes. Our work underscores the necessity of further exploration to enhance transplant viability and highlights the importance of understanding the immunological aspects of transplant acceptance and longevity.

## Data Availability

The original contributions presented in the study are included in the article/**Supplementary Material**. Further inquiries can be directed to the corresponding author.

## Supplementary material

Supplementary Table 1 Total DEGs in Mø-C1.

Supplementary Table 2 Total DEGs in Mø-C2.

Supplementary Table 3 Total DEGs in Mø-C3.

Supplementary Table 4 Up-regulated DEGs in allograft in related pathways.

Supplementary Table 5 Down-regulated DEGs in allograft in related pathways.

Supplementary Table 6 Cytokine Signature Enrichment in Mø-C1.

Supplementary Table 7 Cytokine Signature Enrichment in Mø-C2.

Supplementary Table 8 Cytokine Signature Enrichment in Mø-C3.

## References

[1] Bornstein SR Ludwig B Steenblock C. Progress in islet transplantation is more important than ever. Nat Rev Endocrinol. (2022) 18:389–90. doi: 10.1038/s41574-022-00689-0 PMC9109192 35578026

[2] Harris E. FDA greenlights first cell therapy for adults with type 1 diabetes. JAMA. (2023) 330(5):402. doi: 10.1001/jama.2023.12542 37436739

[3] Mullard A. FDA approves first cell therapy for type 1 diabetes. Nat Rev Drug Discovery. (2023) 22:611. doi: 10.1038/d41573-023-00113-w 37419946

[4] FDA. FDA approves first cellular therapy to treat patients with type 1 diabetes (2023). Available online at: https://www.fda.gov/news-events/press-announcements/fda-approves-first-cellular-therapy-treat-patients-type-1-diabetes (Accessed February 5, 2024).

[5] Marfil-Garza BA Imes S Verhoeff K Hefler J Lam A Dajani K. Pancreatic islet transplantation in type 1 diabetes: 20-year experience from a single-centre cohort in Canada. Lancet Diabetes Endocrinol. (2022) 10:519–32. doi: 10.1016/S2213-8587(22)00114-0 35588757

[6] Chen Q-D Liu L Zhao X-H Liang J-B Li S-W. Challenges and opportunities in the islet transplantation microenvironment: a comprehensive summary of inflammatory cytokine, immune cells, and vascular endothelial cells. Front Immunol. (2023) 14:1293762. doi: 10.3389/fimmu.2023.1293762 38111575 PMC10725940

[7] Li Q Lan P. Activation of immune signals during organ transplantation. Signal Transduct Target Ther. (2023) 8:110. doi: 10.1038/s41392-023-01377-9 36906586 PMC10008588

[8] Wang X Brown NK Wang B Shariati K Wang K Fuchs S. Local immunomodulatory strategies to prevent allo-rejection in transplantation of insulin-producing cells. Adv Sci. (2021) 8:2003708. doi: 10.1002/advs.202003708 PMC8425879 34258870

[9] Weir GC Bonner-Weir S. Scientific and political impediments to successful islet transplantation. Diabetes. (1997) 46:1247–56. doi: 10.2337/diab.46.8.1247 9231647

[10] Zahr E Molano RD Pileggi A Ichii H Jose SS Bocca N. Rapamycin impairs in *vivo* proliferation of islet beta-cells. Transplantation. (2007) 84:1576–83. doi: 10.1097/01.tp.0000296035.48728.28 18165767

[11] Beilke JN Kuhl NR Kaer LV Gill RG Beilke JN Kuhl NR. NK cells promote islet allograft tolerance *via* a perforin-dependent mechanism. Nat Med. (2005) 11:1059–65. doi: 10.1038/nm1296 16155578

[12] Yan L Ye L Chen Y He S Zhang C Mao X. The influence of microenvironment on survival of intraportal transplanted islets. Front Immunol. (2022) 13:849580. doi: 10.3389/fimmu.2022.849580 35418988 PMC8995531

[13] Li X Meng Q Zhang L. The fate of allogeneic pancreatic islets following intraportal transplantation: challenges and solutions. J Immunol Res. (2018) 2018:2424586. doi: 10.1155/2018/2424586 30345316 PMC6174795

[14] Na YR Kim SW Seok SH. A new era of macrophage-based cell therapy. Exp Mol Med. (2023) 55:1945–54. doi: 10.1038/s12276-023-01068-z PMC10545778 37653035

[15] Gou W Wang J Song L Kim D-S Cui W Strange C. Alpha-1 antitrypsin suppresses macrophage activation and promotes islet graft survival after intrahepatic islet transplantation. Am J Transplant. (2021) 21:1713–24. doi: 10.1111/ajt.16342 PMC8082666 33047509

[16] Zhao Y-Z Huang Z-W Zhai Y-Y Shi Y Du C-C Zhai J. Polylysine-bilirubin conjugates maintain functional islets and promote M2 macrophage polarization. Acta Biomater. (2021) 122:172–85. doi: 10.1016/j.actbio.2020.12.047 33387663

[17] Li Y Ding X Tian X Zheng J Ding C Li X. Islet transplantation modulates macrophage to induce immune tolerance and angiogenesis of islet tissue in type I diabetes mice model. Aging (Albany NY). (2020) 12:24023–32. doi: 10.18632/aging.104085 PMC7762494 33221752

[18] Kumar M Gupta P Bhattacharjee S Nandi SK Mandal BB. Immunomodulatory injectable silk hydrogels maintaining functional islets and promoting anti-inflammatory M2 macrophage polarization. Biomaterials. (2018) 187:1–17. doi: 10.1016/j.biomaterials.2018.09.037 30286320

[19] Chen P Yao F Lu Y Peng Y Zhu S Deng J. Single-cell landscape of mouse islet allograft and syngeneic graft. Front Immunol. (2022) 13:853349. doi: 10.3389/fimmu.2022.853349 35757709 PMC9226584

[20] Hao Y Hao S Andersen-Nissen E Mauck WM Zheng S Butler A. Integrated analysis of multimodal single-cell data. Cell. (2021) 184:3573–3587.e29. doi: 10.1016/j.cell.2021.04.048 34062119 PMC8238499

[21] McInnes L Healy J Saul N Großberger L. UMAP: Uniform manifold approximation and projection. JOSS. (2018) 3:861. doi: 10.21105/joss.00861

[22] Korsunsky I Millard N Fan J Slowikowski K Zhang F Wei K. Fast, sensitive and accurate integration of single-cell data with Harmony. Nat Methods. (2019) 16:1289–96. doi: 10.1038/s41592-019-0619-0 PMC6884693 31740819

[23] Hänzelmann S Castelo R Guinney J Hänzelmann S Castelo R Guinney J. GSVA: gene set variation analysis for microarray and RNA-Seq data. BMC Bioinf. (2013) 14:7. doi: 10.1186/1471-2105-14-7 PMC3618321 23323831

[24] Qiu X Mao Q Tang Y Wang L Chawla R Pliner HA. Reversed graph embedding resolves complex single-cell trajectories. Nat Methods. (2017) 14:979–82. doi: 10.1038/nmeth.4402 PMC5764547 28825705

[25] Cui A Huang T Li S Ma A Pérez JL Sander C. Dictionary of immune responses to cytokines at single-cell resolution. Nature. (2024) 625:377–84. doi: 10.1038/s41586-023-06816-9 PMC10781646 38057668

[26] Irac SE Soon MSF Borcherding N Tuong ZK Irac SE Soon MSF. Single-cell immune repertoire analysis. Nat Methods. (2024) 21:777–92. doi: 10.1038/s41592-024-02243-4 38637691

[27] Wang Y Wang J-Y Schnieke A Fischer K Wang Y Wang J-Y. Advances in single-cell sequencing: insights from organ transplantation. Military Med Res. (2021) 8:45. doi: 10.1186/s40779-021-00336-1 PMC8361611 34389057

[28] Mass E Nimmerjahn F Kierdorf K Schlitzer A Mass E Nimmerjahn F. Tissue-specific macrophages: how they develop and choreograph tissue biology. Nat Rev Immunol. (2023) 23:563–79. doi: 10.1038/s41577-023-00848-y PMC10017071 36922638

[29] Kloc M Kubiak JZ. Monocyte and macrophage function diversity. IJMS. (2022) 23:12404. doi: 10.3390/ijms232012404 36293261 PMC9603855

[30] Lendeckel U Venz S Wolke C Lendeckel U Venz S Wolke C. Macrophages: shapes and functions. ChemTexts. (2022) 8:12. doi: 10.1007/s40828-022-00163-4 35287314 PMC8907910

[31] Chung WY Pollard CA Kumar R Drogemuller CJ Naziruddin B Stover C. A comparison of the inflammatory response following autologous compared with allogenic islet cell transplantation. Ann Transl Med. (2021) 9:98–8. doi: 10.21037/atm-20-3519 PMC7867892 33569400

[32] Ravindranath MH El Hilali F Filippone EJ. The impact of inflammation on the immune responses to transplantation: tolerance or rejection? Front Immunol. (2021) 12:667834. doi: 10.3389/fimmu.2021.667834 34880853 PMC8647190

[33] Kloc M Ghobrial RM. Chronic allograft rejection: A significant hurdle to transplant success. Burns Trauma. (2014) 2:3–10. doi: 10.4103/2321-3868.121646 27574640 PMC4994504

[34] Chen Y-P Yin J-H Li W-F Li H-J Chen D-P Zhang C-J. Single-cell transcriptomics reveals regulators underlying immune cell diversity and immune subtypes associated with prognosis in nasopharyngeal carcinoma. Cell Res. (2020) 30:1024–42. doi: 10.1038/s41422-020-0374-x PMC7784929 32686767

[35] Wang S Leng L Wang Q Gu Y Li J An Y. A single-cell transcriptome atlas of human euploid and aneuploid blastocysts. Front Immunol. (2024) 15:1468–81. doi: 10.1038/s41588-024-01788-6 38839885

[36] Huang SSY Toufiq M Eghtesady P Van Panhuys N Garand M Huang SSY. The molecular landscape of sepsis severity in infants: enhanced coagulation, innate immunity, and T cell repression. Front Immunol. (2024) 15:1281111. doi: 10.3389/fimmu.2024.1281111 38817614 PMC11137207

[37] Su EY Fread K Goggin S Zunder ER Cahan P. Direct comparison of mass cytometry and single-cell RNA sequencing of human peripheral blood mononuclear cells. Sci Data. (2024) 11:559. doi: 10.1038/s41597-024-03399-6 38816402 PMC11139855

[38] Walzer KA Tandel J Byerly JH Daniels AM Gullicksrud JA Whelan EC. Transcriptional control of the Cryptosporidium life cycle. Nature. (2024) 630:174–80. doi: 10.1038/s41586-024-07466-1 PMC12057246 38811723

[39] Wu J Li W Su J Zheng J Liang Y Lin J. Integration of single-cell sequencing and bulk RNA-seq to identify and develop a prognostic signature related to colorectal cancer stem cells. Sci Rep. (2024) 14:12270. doi: 10.1038/s41598-024-62913-3 38806611 PMC11133358

[40] Merli P Caruana I De Vito R Strocchio L Weber G Bufalo FD. Role of interferon-γ in immune-mediated graft failure after allogeneic hematopoietic stem cell transplantation. Haematologica. (2019) 104:2314–23. doi: 10.3324/haematol.2019.216101 PMC6821635 30792213

[41] Halawi A El Kurdi AB Vernon KA Solhjou Z Choi JY Saad AJ. Uncovering a novel role of focal adhesion and interferon-gamma in cellular rejection of kidney allografts at single cell resolution. Front Immunol. (2023) 14:1139358. doi: 10.3389/fimmu.2023.1139358 37063857 PMC10102512

[42] Fueyo-González F McGinty M Ningoo M Anderson L Cantarelli C Angeletti A. Interferon-β acts directly on T cells to prolong allograft survival by enhancing regulatory T cell induction through Foxp3 acetylation. Immunity. (2022) 55:459–474.e7. doi: 10.1016/j.immuni.2022.01.011 35148827 PMC8917088

[43] Boieri M Ulvmoen A Sudworth A Lendrem C Collin M Dickinson AM. IL-12, IL-15, and IL-18 pre-activated NK cells target resistant T cell acute lymphoblastic leukemia and delay leukemia development. vivo OncoImmunol. (2017) 6:e1274478. doi: 10.1080/2162402X.2016.1274478 PMC5384344 28405496

[44] Wu S Ma R Zhong Y Chen Z Zhou H Zhou M. Deficiency of IL-27 signaling exacerbates experimental autoimmune uveitis with elevated uveitogenic th1 and th17 responses. IJMS. (2021) 22:7517. doi: 10.3390/ijms22147517 34299138 PMC8305313

[45] Ashour D Arampatzi P Pavlovic V Förstner KU Kaisho T Beilhack A. IL-12 from endogenous cDC1, and not vaccine DC, is required for Th1 induction. JCI Insight. (2020) 5:e135143. doi: 10.1172/jci.insight.135143 32434994 PMC7259537

[46] Zhou H Pu Z Lu Y Zheng P Yu H Mou L. Elucidating T cell dynamics and molecular mechanisms in syngeneic and allogeneic islet transplantation through single-cell RNA sequencing. Front Immunol. (2024) 15:1429205. doi: 10.3389/fimmu.2024.1429205 39100662 PMC11294159

